# Ocular tissue distribution in the State of São Paulo: analysis on corneal discarding reasons^*^

**DOI:** 10.1590/1518-8345.3041.3196

**Published:** 2019-10-14

**Authors:** João Luis Erbs Pessoa, Janine Schirmer, Denise de Freitas, Neide da Silva Knihs, Bartira de Aguiar Roza

**Affiliations:** 1 Secretaria de Saúde do Estado de São Paulo, Central de Transplantes, São Paulo, SP, Brasil.; 2 Universidade Federal de São Paulo, Escola Paulista de Enfermagem, São Paulo, SP, Brasil.; 3 Universidade Federal de São Paulo, Escola Paulista de Medicina, São Paulo, SP, Brasil.; 4 Universidade Federal de Santa Catarina, Florianópolis, SC, Brasil.

**Keywords:** Tissue and Organ Procurement, Corneal Transplantation, Tissue Banks, Tissue and Organ Harvesting, Tissue Donors, Nursing, Obtenção de Tecidos e Órgãos, Transplante de Córnea, Banco de Tecidos, Coleta de Tecidos e Órgãos, Doadores de Tecidos, Enfermagem, Obtención de Tejidos y Órganos, Trasplante de Córnea, Bancos de Tejidos, Recolección de Tejidos y Órganos, Donantes de Tejidos, Enfermería

## Abstract

**Objective:**

to identify the reasons for refusal of corneas.

**Method:**

this was a cross-sectional, retrospective, descriptive and correlational study composed of 5,560 optical corneas. The information was taken from the notification, organ procurement and distribution centers database as well as donor records. Descriptive statistics were used for the analysis of categorical variables and specific tests with a significance level of 5% for assessing the associations between variables. This study met the ethical aspects of scientific research.

**Results:**

60% of the donors were male and 40% died by circulatory problems. The main reason for refusal as informed by transplant teams is the donor’s age and the endothelial cell count. For each year added to the donor’s age, there is a 1% decrease in the chance that this cornea will be used for transplantation, and the increase of 100 cells per mm2 increases the chances that this cornea will be used by 9%.

**Conclusion:**

the main cause of refusal in the acceptance of corneal tissue is related to the age and the endothelial cell count.

## Abstract (pt)

**Objetivo:**

identificar os motivos da recusa de córneas.

**Método:**

estudo transversal, retrospectivo, descritivo e correlacional, composto por 5.560 córneas ópticas. As informações foram extraídas do banco de dados da Central de Notificação, Captação e Doação de Órgãos (CNCDO), bem como de prontuários de doadores. A estatística descritiva foi utilizada para a análise das variáveis categóricas e testes específicos, com nível de significância de 5% para avaliar as associações entre as variáveis. Este estudo atendeu aos aspectos éticos da pesquisa científica.

**Resultados:**

60% dos doadores eram do sexo masculino e 40% morreram por problemas circulatórios. Os principais motivos de recusa informados pelas equipes de transplante são a idade do doador e a contagem de células endoteliais. Para cada ano adicionado à idade do doador, há uma redução de 1% na chance de que essa córnea seja usada para transplante, e para cada acréscimo de 100 células por mm2 aumenta as chances de que essa córnea seja usada em 9%.

**Conclusão:**

a principal causa de recusa na aceitação do tecido corneano está relacionada à idade e à contagem de células endoteliais.

## Abstract (es)

**Objetivo:**

identificar los motivos para el rechazo de córneas.

**Método:**

estudio transversal, retrospectivo, descriptivo y correlacional, compuesto por 5.560 córneas ópticas. La información se obtuvo de la bases de datos de Centros de Notificación, Obtención de Órganos y Distribución, así como de los registros de los donantes. Se utilizaron estadísticas descriptivas para analizar las variables categóricas y pruebas específicas con un nivel de significación de 5%, para evaluar las asociaciones entre variables. Este estudio contempla los aspectos éticos de una investigación científica.

**Resultados:**

el 60% de los donantes eran hombres y el 40% falleció por problemas circulatorios. La principal razón del rechazo, informada por los equipos de trasplante, es la edad del donante y el recuento de las células endoteliales. Por cada año agregado a la edad del donante, existe una disminución de 1% en la probabilidad de que la córnea pueda ser utilizada para trasplante; el aumento de 100 células por mm2 aumenta la posibilidad de que esta córnea será usada por 9%.

**Conclusión:**

la principal causa de rechazo en la aceptación del tejido corneal está relacionada con la edad y el recuento de células endoteliales.

## Introduction

Corneal diseases are the third cause of blindness worldwide, after the cataract and the glaucoma. Currently, more than 10 million people suffer from bilateral corneal diseases. Over 53% of the world’s population does not have access to corneal transplantation^1^.

Brazil had over 5.379 patients in the waiting list for a cornea transplantation in 2013, and 13.744 procedures were carried out that year^2^. At the end of 2017 the number of patients in the waiting list was 9.266 and the number of transplants carried out was 15.242^3^. According to the National Transplant System, in 2016, the average waiting time was of 6.7 months. In 2015, the average waiting time for an optical corneal in the State of São Paulo was 4.7 months^4^. Promoting compatible organ and tissues with the number of patients waiting for a transplant is one of the main difficulties faced by the National Transplant System.

There is still a great number of underreported cornea donors considering the number of deaths in health institutions (1,227,039 deaths/2014)^2^ and the possibility of these patients become effective donors. There are States and municipalities in Brazil with a greater number of donors and, therefore, with more cornea offers to transplantation centers.

The number of corneal transplants carried out in Brazil is lower than expected, considering the number of corneas retrieval. There is no statistical data that may reveal the exact number of corneas reported, considering the overall number of deaths, not even a record of discarded corneas.

The main layers of the cornea are: epithelium, bowman’s layer, stroma, Descemet Membrane and endothelium. In 2016 the estimate demand of corneal transplants was 18,401, however, only 14,534 were performed. This difference between the need for transplantation and what is performed has generated an increase in the number of patients waiting for this procedure, from 2013 to 2016^3^. The main reasons for the discarding of the collected corneas are: factors associated with morphological quality of donated corneas and serological tests. The ocular tissue banks perform the biomicroscopic examination using a slit lamp apparatus to assess the quality of the cornea donor. This evaluation criterion is based on scores of zero -four on the following questions: intact epithelium; senile halo; stromal edema; Descemet folds; Guttata and endothelial density. Grade zero is considered excellent, grade one is good, grade two is regular, grade three is bad and grade four is the worst, considered unacceptable.

In general, corneas that receive grades between zero and one on evaluated items and have more than 2,000 cells per square millimeter are considered optical. From this evaluation, the corneal tissue receives an optical or tectonic classification. It is worth notincing that not all eye banks perform endothelial cell counts and, in these cases, this evaluation follows subjective biomicroscopic parameter. Corneas classified as optic can be transplanted for the purpose of reestablishing or improving the receptor’s vision. The tissue evaluated as tectonic has the objective of preserving corneal anatomy and integrity in situations of surgical emergency of the receptor.

Thus, we understand how fundamental is to investigate factors that may trigger the loss of eye tissue in Brazil. With these information, governmental and non-governmental authorities can develop improvement strategies that affect this scenario, in addition to enhance the quality of tissues offered to transplantation teams as well increase patient safety. Hence, the guiding questions of this study are: “What are the causes of ocular tissue refusal and how to increase the supply of quality tissues to corneal transplants?”.

The contribution of this study is heavily based on improvements in the transplant scenario in Brazil, in addition to strengthening and expanding the topic of cornea transplant in the country, improving performance, care and storage of ocular tissue, which are important academic and scientific advances in the field. Thus, the aim of this study is to identify the causes of refusal of ocular tissues collected in the State of São Paulo, Brazil.

## Method

This is a cross-sectional, retrospective, correlational and descriptive study about the disposal of corneas collected and released for transplant, in which we analyzed variables related to the quality of this tissue. The charts of ocular tissue donors (corneas) containing information on the biomicroscopic data and the classification of the tissues provided by ocular tissue banks for the transplant center in the State of São Paulo were used. Data from the distribution process of these tissues to potential corneal receptors were used and these data were taken from the software database of the State System of Transplant Management System (SIGSET).

The population was composed by an analysis of all donor records obtained in the State of São Paulo, in 2013, comprising 12,290 corneas evaluated as optics and available from tissue banks for distribution and allocation. Tectonic corneas were not included in this study and the sample was composed of 5,560 corneas. Data collection script included [1] donordemographic variables (city where death occurred, domicile, age, sex, etc.), [2] retrieval andpreservation of tissue (time between death and enucleation, time between death andpreservation, and information on body cooling), [3] cornea quality (epithelium, halo, edema, Descemet’s Membrane folds, guttata, density and cell count), [4] distribution and [5] refusal formed by transplantation teams. All the optic corneas available in the State of São Paulo for transplantation were analyzed. In order to collect data, a team of professionals with technical knowledge was trained to extract SIGSET information from CNCDO-SP (Center for Notification, Collection and Distribution of Organs and Tissues- São Paulo State), besides analyzing the medical records of the donor. The information collected was inserted in Excel^®^ worksheet, composing the database of this research.

For the statistical analysis, tests for descriptive review of association and logistic regression were used. The linear associations between two variables of numerical nature were evaluated by Pearson’s correlation (between cell count (mm2) and donor age, time between death and enucleation, time between death and preservation).For all statistical tests, a significance level of 5% using SPSS 20.0and Stata 12 was used. This study was approved by the Research Ethics Committee of *Universidade Federal de São Paulo* (UNIFESP) under the protocol number of the Certificate of Presentation for Ethical Appreciation 31450414.4.0000.5505.

## Results

Out of the 5,560 corneas evaluated, 60.2% came from male donors, with an average age of 53 years (median 56, minimum two, maximum 80, first quartile 42 and third quartile 66) and 40.3% of corneas came from donors whose cause of death was related to circulatory system diseases.

The average time between death and enucleation was 4.3 hours (standard deviation of 3.4 hours), and the average time between death and preservation was 10.2 hours (standard deviation of 5.5 hours). Harvested corneas showed the following averages in the evaluations: intact epithelium 1; senile halo 1; stromal edema 1; the Descemet’s Membrane folds 1; endothelial density 1; guttata 0 and mean of 2,492 cells. Out of these, 80% were accepted and transplanted. Transplanted corneas showed the following averages in the evaluations: intact epithelium 1; senile halo 0; stromal edema 0; the Descemet’s Membrane folds 1; endothelial density 0; guttata 0 and mean of 2,514 cells.

The main causes of refusal informed by transplantation teams at the time of the offer of the corneas were the quality of the cornea (35.2%), team in another procedure (28%), long distance to remove the cornea (19.2%), too long preservation time (6.1%), other causes (11.5%). On average, each cornea had 9.3 refusals before being used or disposed of.

There was an association between transplantation and age (p<0.001) and cause of death (p<0.001), since corneas from donors between 15 and 49 years presented higher transplant (acceptance) percentages than corneas whose donors were older than 50 years. Corneas from donors who have died by external causes (multiple trauma, head trauma, gunshot wound, traffic accident, drowning, exogenous intoxication, etc.) had the highest percentages of transplant.

Table 1 shows an association between all the variables of cornea quality and transplantation (p<0.001), so that the corneas of the donors whose body was preserved (cold storage) that showed values of zero for senile halo, stromal edema, endothelial density and guttate and values of one for epithelium and Descemet’s Membrane folds had higher acceptance percentages regarding transplantation.

**Table 1:** – Distribution of optical corneas by quality and use for transplant. São Paulo, SP, Brazil, 2016

Corneal evaluation	Transplantation				Total		p^**‡**^
	Yes*		No^**†**^				
	N	%	N	%	N	%	
*Preserved body*	4.418	80%	1.104	20%	5.522	100%	0,011
*Yes*	1.912	81,6%	431	18,4%	2.343	100%	
							
*Intact epithelium*	4.418	80%	1.104	20%	5.522	100%	0,049
1.0	3.291	81%	774	19%	4.065	100%	
							
*Senile halo*	4.416	80%	1.104	20%	5.520	100%	<0,001
0	1.238	84,8%	222	15,2%	1.460	100%	
							
*Stromal edema*	4.417	80%	1.102	20%	5.519	100%	<0,001
0	832	87,8%	116	12,2%	948	100%	
							
*Descemet’s membrane folds*	4.418	80%	1.104	20%	5.522	100%	<0,001
1.0	1.972	82,9%	408	17,1%	2.380	100%	
							
*Endothelial density*	4.353	79,9%	1.092	20,1%	5.445	100%	0,103
0	1.024	81,9%	226	18,1%	1.250	100%	
							
*Guttata*	4.416	80%	1.103	20%	5.519	100%	0,003
0	2.555	81,3%	587	18,7%	3.142	100%	

*Yes = transplanted corneas; ^†^No = refused corneas; ^‡^p = description level of the Chi-square or Fisher’s exact test

We can see in table 2 that transplanted corneas showed lower average donor age and increased endothelial cell count. In the logistic regression model, it was possible to identify that, for each one-year increase in donor age there is a 1% reduction in the odds of the cornea being accepted for transplantation (p<0.001). Corneas from donors who have died because of nervous system diseases are 46% less likely to be transplanted (p=0.016).

**Table 2:** Donor age, time between death and enucleation, time between death and preservation and cell count by transplant status, São Paulo, SP, Brazil, 2016

Variables	Mean	Standard deviation	Minimum	Maximum	1st quartile	Median	3rd quartile	N^‡^	p^§^
Donor age (years)									<0,001
Yes*	51,93	17,40	2,00	80,00	41,00	55,00	65,00	4.418	
No^†^	56,03	15,54	2,00	80,00	48,25	58,00	68,00	1.104	
									
Time between death and enucleation (hours)									0,074
Yes*	4,32	3,41	0,08	29,17	2,17	3,50	5,17	4.418	
No^†^	4,12	3,19	0,08	49,58	2,25	3,38	5,00	1.104	
									
Time between death and preservation (hours)									0,519
Yes*	10,21	5,51	0,42	42,00	5,83	9,50	13,69	4.418	
No^†^	10,09	5,51	0,67	49,92	5,67	9,25	13,67	1.104	
									
Cells (mm^2^)									<0,001
Yes*	2.514,50	694,69	1.831,00	3.968,00	2.288,00	2.481,00	2.680,00	4.293	
No^†^	2.405,45	280,58	1.834,00	3.617,00	2.188,00	2.368,00	2.590,00	1.076	

*Yes = transplanted corneas; ^†^No = refused corneas; ^‡^N = Yes; 4,418/ No = 1,104; ^§^p = description level of Chi-square or Fisher’s exact test

In addition, corneas evaluated with a three score regarding senile halo are 85% less likely of being transplanted if compared with corneas evaluated with better values (p=0.015). Corneas with a value of zero concerning stromal edema are 65% more likely to be transplanted if compared with the corneas that had other values. On the other hand, this chance is 29% lower for those that received score dois (p<0.001).

According to the logistic regression model, corneas that had a dois score regarding endothelial density are 23% less likely to being transplanted (p<0.001). For each 100-cell increase *per* mm^2^ in offered corneas there is an increase of 9% in the chance of transplantation (p<0.001).

Furthermore, in the negative binomial regression model, it was observed that for corneas that received score 0 regarding intact epithelium have 29% less refusals than those classified with other scores (p=0.004). Corneas with score zero regarding senile halo are 39% less likely to be refused than those with higher scores (p<0.001). For corneas with stromal edema scored zero, there is a 35% lower refusal compared with those which received score two and are 43% greater chance of refusal (p<0,001).

Corneas that had a score two concerning Descemet’s membrane folds have 17% more refusals (p=0.004). For each 100-cells increase *per* mm^2^ there is a 15% reduction in the average number of refusals (p<0.001).

## Discussion

The results reveal similarity regarding gender and cause of death of the organ and tissue donors. In 2014, data from the Ministry of Health showed that 56.5% of deaths were related to male. The main cause of death was also related to circulatory system diseases, with 27.7%, followed by the neoplastic diseases, with 16.4%^5-6^. Other studies have found a higher number of male donors^7-14^. Out of 2,854 effective donors in 2015 in Brazil, 59% were male^15^.

In this research, we observed that 63% of donors were over 50 years, considering that there is a 80-year-old limit to donations, as determined by the Portaria 2.600, of October 21st 2009^16^. The literature shows that when there are more cornea donors than receptors, transplantation teams tend to choose corneas from younger donors^17^.

The main causes of refusal or dispose of ocular tissue found in this study were related to cornea quality (35,2%), unavailability of the team for being in another procedure (28%) and long distance to remove the cornea (19.2%). According to data from the National Health Surveillance Agency, 12% of the total collected eyeballs in Brazil were disposed of due to poor quality in 2014^18^. A research with Canadian corneal transplant doctors found that donor quality is one of the contributing factors to increase the waiting time for corneal transplants^19^.

In our study it was possible assess a correlation between transplant, donor age and cause of death, and that younger donors ranging from 15 to 49 years and whose cause of death was associated with external causes had a higher percentage of transplant (p<0.001). It is worth mentioning that these donors had a higher percentage of endothelial cell count when compared with other donors.

Another study corroborates our findings showing that donors whose cause of death was related to external causes had a higher average of endothelial cell count^20^. Other studies have shown statistically significant association between increased donor age and decreased endothelial cell density^7-8,10,21-22^.

A study carried out in an eye bank showed that corneas from donors who were aged between 20 and 29 years had higher percentages of classification as optics^14^. Endothelial density reduction caused by the advancement of donor age reduces the likelihood of these older donor corneas to be used for transplants^8^. However, many corneas of donors over 80 years old have quality to be transplanted.

Donors whose cause of death was trauma have better cornea quality, when compared with corneas from donors with other causes of death^23^. When there are more cornea donors thanreceptors, transplant doctors tend to choose corneas from donors whose death was acute trauma-related^17^.

Cornea donors whose cause of death was trauma were 50% less likely to show graft failure when compared with corneas from donor who died from other causes^24^. Some diseases such as Diabetes Mellitus and history of cataract surgery, affect the quality of the cellular density of the endothelium^22^.

Chi-square, Fisher’s exact and Student’s t tests (p<0.001) showed that corneas with a higher quality, lower donor mean age, preservation of the donor’s body (cold chamber) and greater number of endothelial cells had a higher percentage of acceptance for transplantation. Thus, the lower the score received in all the evaluated items by the ocular tissue bank and the greater the number of endothelial cells, the faster the cornea will be accepted for transplantation.

With the regression model, it was possible to identify that age is a relevant factor to the acceptance or refusal of the cornea offered. However, there are many studies that have proven that donor age does not interfere or influence with the result of the transplant^24-27^.

However, endothelial density tends to decrease with age. Under normal conditions, for healthy individuals endothelial cell density decreases at a rate of 0.5% to 0.6% each year. Under corneal transplant conditions, the loss of these cells is more enhanced. The minimum quantity of endothelial cells needed to keep the endothelium working is 500 cells *per* mm^2^.

In most corneal transplantation teams in the State of São Paulo, the determination of acceptance or rejection is strictly linked to the number of endothelial cells that the donor has. Thus, the age of the donor, that is a factor that alters the quality of the endothelial tissue, decreases the probability of the use for the transplant.

The experience of The United States of America (USA) eye banks reported that when choosing corneas for transplant, surgeons tend to create more restrictive parameters, preferring corneas with the largest number of endothelial cells and from younger donors^28^.

When using logistic regression models or negative binomial regression for senile halo, stromal edema, endothelial density, intact epithelium and Descemet’s Membrane folds, it was possible to identify with statistical significance, that well-evaluated corneas regarding these issues have greater chances of being accepted for transplantation. Therefore, poorly-evaluated corneas are more likely to be rejected by transplantation teams. We did not find other studies that deal with distribution and allocation of corneal tissue.

## Conclusion

The main reasons for refusal or disposal informed by transplantation teams are related to the quality of corneas offered by the Transplant Center. It was observed that corneas from donors whose body was in cold storage were less rejected. Corneas from younger donors whose cause of death was related to external causes had a higher percentage of use for transplants. Similarly, for each increase of 100 cells *per* mm^2^ on donor corneas, there is an increase of 9% of this cornea being used for transplantation and a reduction of 1% on average of refusals. For each one-year increase in donor age, there is a reduction of 1% in the chance of the cornea being accepted for transplant.

As assessed, quality is one of the major factors associated with refusal, and the quality is greater in younger donor corneas. One way of improving tissue quality and thus lowering the refusal would be to limit donor age, which is 80 years in the State of São Paulo. However, it is worth mentioning that the decision of reducing donor age to increase tissue quality requires a careful analysis of the number of patients waiting for a transplant and the number of corneas provided. States where the waiting list (technical registration) has many receptors, with a positive variation, it is not advisable to restrict donor age, because although it increases the quality of the tissue retrieval, it can decrease the number of corneas available. It is the State’s responsibility, considering the high number of corneas refused by the distance that the transplantation team will have to go through to gain access to the tissue, to create ways that allow this cornea to reach the transplantation team.

The most important limitation of this study is the fact that transplantation clinical follow-up data was not included, correlating the success or failure of the transplant with the quality of the corneas offered. Because this is an observational and retrospective study, it is not possible to establish a cause and effect relationship.
